# Alcohol and the risk for latent autoimmune diabetes in adults: results based on Swedish ESTRID study

**DOI:** 10.1530/EJE-14-0403

**Published:** 2014-11

**Authors:** Bahareh Rasouli, Tomas Andersson, Per-Ola Carlsson, Mozhgan Dorkhan, Valdemar Grill, Leif Groop, Mats Martinell, Tiinamaja Tuomi, Sofia Carlsson

**Affiliations:** 1 Epidemiology Unit, Institute of Environmental Medicine (IMM), Karolinska Institutet, SE 171 77, Stockholm, Sweden; 2 Center for Occupational and Environmental Medicine, Stockholm County Council, Stockholm, Sweden; 3 Department of Medical Sciences, Uppsala University, SE-751 85, Uppsala, Sweden; 4 Department of Clinical Sciences in Malmö, Clinical Research Centre, Lund University, SE-205 02, Malmö, Sweden; 5 NTNU Institute of Cancer Research and Molecular Medicine, Norwegian University of Science and Technology, Trondheim University Hospital, Trondheim, Norway; 6 Department of Public Health and Caring Sciences, Uppsala University, SE-751 22, Uppsala, Sweden; 7 Division of Endocrinology, Department of Medicine, Helsinki University Central Hospital, Research Program for Diabetes and Obesity and Folkhalsan Research Center, University of Helsinki, F-00014, Helsinki, Finland

## Abstract

**Objective:**

Moderate alcohol consumption is associated with a reduced risk of type 2 diabetes. Our aim was to investigate whether alcohol consumption is associated with the risk of latent autoimmune diabetes in adults (LADA), an autoimmune form of diabetes with features of type 2 diabetes.

**Design:**

A population-based case–control study was carried out to investigate the association of alcohol consumption and the risk of LADA.

**Methods:**

We used data from the ESTRID case–control study carried out between 2010 and 2013, including 250 incident cases of LADA (glutamic acid decarboxylase antibodies (GADAs) positive) and 764 cases of type 2 diabetes (GADA negative), and 1012 randomly selected controls aged ≥35. Logistic regression was used to estimate the odds ratios (ORs) of diabetes in relation to alcohol intake, adjusted for age, sex, BMI, family history of diabetes, smoking, and education.

**Results:**

Alcohol consumption was inversely associated with the risk of type 2 diabetes (OR 0.95, 95% CI 0.92–0.99 for every 5-g increment in daily intake). Similar results were observed for LADA, but stratification by median GADA levels revealed that the results only pertained to LADA with low GADA levels (OR 0.85, 95% CI 0.76–0.94/5 g alcohol per day), whereas no association was observed with LADA having high GADA levels (OR 1.00, 95% CI 0.94–1.06/5 g per day). Every 5-g increment of daily alcohol intake was associated with a 10% increase in GADA levels (*P*=0.0312), and a 10% reduction in homeostasis model assessment of insulin resistance (*P*=0.0418).

**Conclusions:**

Our findings indicate that alcohol intake may reduce the risk of type 2 diabetes and type 2-like LADA, but has no beneficial effects on diabetes-related autoimmunity.

## Introduction

Recent findings from the Norwegian HUNT study have suggested that moderate intake of alcohol is associated with a reduced risk of autoimmune diabetes in adults (1). However, these findings were based on a limited number of cases and the role of sex, beverage preferences, dose–response, and underlying mechanisms could not be addressed. Further investigations and replications are thus needed.

Latent autoimmune diabetes in adults (LADA) is estimated to account for 9% of all diabetes in Europe according to a recent report, making it the second most common form of diabetes (2). Compared with classical type 1 diabetes, progression of autoimmune β-cell failure occurs slowly in LADA (2, 3), and insulin treatment is often not required at the time of diagnosis. Sometimes termed diabetes 1.5, LADA also has features of type 2 diabetes, including insulin resistance (IR) (4). It is appreciated that similarities with type 1 (degree of autoimmunity) and type 2 diabetes (degree of IR) are variable between patients, attesting to heterogeneity of LADA.

Numerous studies have shown that moderate alcohol consumption is inversely associated with type 2 diabetes (5, 6). A potential protective effect has been attributed to improvement in insulin sensitivity (7), and reduction of inflammatory process (8). In addition, moderate alcohol consumption has been associated with a reduced risk of some autoimmune disorders such as rheumatoid arthritis (9, 10) and Graves' hyperthyroidism (11). As an underlying mechanism, it has been suggested that alcohol can exert effects on the modulating immune function and regulate proinflammatory molecules (8, 12).

Against this background, we hypothesized that alcohol may prevent or delay the onset of LADA, either through beneficial effects of alcohol on insulin sensitivity or through effects on autoimmunity. Our aim was to investigate alcohol consumption and the risk of LADA using data from the largest population-based study of LADA to date (ESTRID; Epidemiological study of risk factors for LADA and type 2 diabetes) with specific focus on the dose–response relation, beverage choice, frequency of alcohol intake, and degree of autoimmunity as assessed by antibody level, glutamic acid decarboxylase antibodies (GADAs).

## Subjects and methods

### Study population and design

This study was based on data from ESTRID, a new case–control study using incident cases of LADA and type 2 diabetes (13). Cases are recruited through recently launched diabetes registries in two Swedish counties covering ∼1 600 000 inhabitants: Scania and Uppsala. These registries are aimed at characterizing all new cases of diabetes according to diabetes type, clinical features, and genetic factors (All New Diabetics in Scania (ANDIS), http://andis.ludc.med.lu.se and All New Diabetics in Uppsala (ANDIU), http://www.andiu.se/). For ESTRID, we invited all incident cases of LADA identified in Scania (2010–) and in Uppsala (2012–), together with a random sample of type 2 diabetes cases (four per LADA case). Controls without diabetes and aged ≥35 years (six per LADA case) were randomly selected from the population of Scania and Uppsala matching for date of participation and residential area (incident density sampling) (14). Participation was high among both cases (80.2%) and controls (66.5%). ESTRID is an ongoing study, and eligible for the analysis of this study were all cases and controls collected and computerized from 1st September 2010 to 1st June 2013 with complete information on alcohol consumption and covariates, including 250 cases of LADA, 764 cases of type 2 diabetes, and 1012 controls. Ethical permission was obtained from the ethical review board in Stockholm and all participants provided written informed consent.

We have previously reported a reduced risk of LADA in relation to alcohol based on the Norwegian HUNT study (1). Owing to the limited number of cases in this study, we did not perform analysis by GADAs. In the present report, we reanalyzed the HUNT data stratified by median GADA levels. HUNT is a large population-based prospective study, in which all inhabitants, aged 20 years or older, of Nord-Trøndelag in Norway were investigated in three consecutive studies during the period of 1984–2008. Among self-reported diabetes patients, presence of GADAs and age at onset of ≥35 years were used to identify incident cases of adult-onset autoimmune diabetes (*n*=65 with low GADA levels and *n*=57 with high GADA levels). Hazard ratios (HRs) of autoimmune diabetes in low and high GADA groups in relation to frequency of alcohol consumption (abstainers, <1, 1–4, and ≥5 times over the last 14 days) were estimated by Cox regression in the present study.

### Biochemical analysis and identification and classification of cases

Cases of diabetes were identified and diagnosed within the health care system in Scania and Uppsala. At diagnosis, blood samples were collected and sent to the university hospital of each county for analysis. GADAs were analyzed by ELISA according to the manufacturer's instructions and the values reported as an index value in relation to standard serum, a level of above 10 IU/ml was regarded as positive according to the instructions in the kit; the method gives a maximum value of 250 IU/ml (15). At cut-off level of 10.7 IU/ml, sensitivity was 84% and specificity was 98%, when GADAs were measured in Ca^2^^+^-treated plasma (15). C-peptide was measured by an IMMULITE 2000 (Siemens Healthcare Diagnostics Product Ltd, Llanberis, UK) or by the Cobas e 601 analyzer (Roche Diagnostics) (16). Based on these measurements, cases with age at onset of ≥35 years were classified as LADA if they were GADA positive (>10) with C-peptide ≥0.2 nmol/l (IMMULITE)/or ≥0.3 (Cobas e 601), and as type 2 diabetes, if they were GADA negative (<10) with C-peptide >0.6 nmol/l (IMMULITE)/or ≥0.72 (Cobas e 601). There is no established definition of LADA but three clinical criteria are commonly used: i) presence of at least one islet autoantibody, most often a GADA, which has the highest penetration, being present in 70–80% of patients with autoimmune diabetes (17); ii) adult age at onset; and iii) insulin independency at diagnosis (18). In this study, the insulin criteria was replaced by C-peptide levels, which provide a more direct measure of remaining insulin production and a slow ‘latent’ onset than insulin treatment, which is open to subjectivity. The homeostasis model assessment (HOMA2) indices for IR (HOMA2-IR) and beta cell function (HOMA2-%B) were obtained using HOMA2 calculator version 2.2.3 freely downloaded from the Oxford Centre for Diabetes, Endocrinology and Metabolism website (19), based on the relationship between fasting plasma glucose and C-peptide levels.

### Assessment of alcohol consumption

The ESTRID questionnaires contained detailed questions on health and lifestyle factors including a previously validated food frequency questionnaire for alcohol intake (20). Cases received it in proximity to diagnosis with careful instructions to report their lifestyle as it was before diagnosis to minimize the impact of diagnosis on lifestyle.

Participants were asked about their alcohol consumption during the preceding year. Among alcohol consumers, data on light and strong beer, cider, red/white/rose wine, liquor, and hard liquor were collected with a question as to how many times the participants usually consumed each type of drink. The nine frequency-response categories ranged from ‘never’ to ≥3 times/day. In addition, information about amount (given in centiliters (cl) for each unit of can, bottle, or glass) of beer, cider, wine, liquor, and hard liquor consumed on each occasion was collected with an open-ended question. To quantify the average alcohol consumption per day, we combined information about the amount and frequency of drinking. The estimated contents of pure alcohol per cl alcoholic beverages were: 0.25 g for light beer, 0.40 g for strong beer, 0.40 g for cider, 0.85 g for wine, 1.5 g for liquor, and 3 g for hard liquor. The questionnaire also contained questions about lifetime alcohol abstention and former drinking.

Based on total alcohol consumption, participants were classified into five groups: non-drinkers, and consumers of 0.01–4.9, 5–14.9, 15–24.9, and ≥25 g alcohol/day. A standard drink contains ∼12 g of alcohol. To avoid sick quitter bias, we used the lowest drinking category as a reference rather than abstainers (21). Frequency of alcohol consumption was classified into five types: ≤1 time/month (as reference group), 2–3 times/month, 1–2 times/week, 3–6 times/week, and ≥1 time/day.

### Statistical analysis

Logistic regressions were used to calculate odds ratios (ORs) for LADA and type 2 diabetes associated with alcohol consumption with 95% CI (SAS 9.3; SAS Institute, Cary, NC, USA). Conditional analyses were also performed by matching for date of participation and residential area. We only present results from the unmatched analyses as these were in close agreement with those from the matched analyses, but allowed us to use all available information. ORs were interpreted as incidence rate ratios, as this case–control study was based on incident cases and controls were sampled by incidence density sampling (14, 22, 23).

To model potential dose–response relationship between total grams of alcohol consumption per day and risk of LADA and type 2 diabetes, we used restricted cubic spline regression (Stata version SE13.0, College Station, TX, USA), which is a flexible way of modeling continuous variables, which allows fitting of smooth and non-linear curves (24). Splines modeled with three knots were used (0.6, 6.5, and 23.0 g/day, selected based on tertiles of alcohol consumption) and participants with the highest alcohol intake of 5% (≥200 g/day) were excluded to reduce the impact of outliers.

The association between alcohol consumption (g/day) and HOMA indices (lnHOMA-IR and lnHOMA-β, logarithmic transformation was applied due to skewing of variables) was assessed using the linear regression model. The association with GADAs (log transformed) was assessed using the Tobit regression model to account for the fact that the outcome variable was truncated at 250 (25). Confounding was adjusted for by inclusion of age, sex, smoking (never, former, and current smokers), BMI (calculated as weight (kg)/height (m^2^), continuous), family history of diabetes (yes/no), and education (primary school, upper secondary school, or university) in all models, unless otherwise specified.

## Results

### Characteristics

Mean age was 60±12 years for LADA patients, 63±10 years for patients with type 2 diabetes, and 59±13 years for controls (Table 1). Compared with controls, patients with LADA and type 2 diabetes were older, heavier, and less physically active. In addition, the proportion of individuals with a family history of diabetes and non-drinkers were higher among patients with diabetes. The median duration of diabetes was 6.2 months in patients with type 2 diabetes and 7.9 months in LADA patients. Duration of diabetes was not related to GADA levels (*P*=0.3978). Patients with type 2 diabetes tended to be older and more likely to be overweight, physically inactive, and with low educational level than LADA patients. LADA patients had lower levels of HOMA-β, and they were more likely to be insulin treated (39 vs 5%). Stratification of LADA patients by median GADA levels (152 IU/ml) indicated that those in low GADA group were older, heavier, had higher levels of C-peptide, had better β-cell function, and were less often treated with insulin compared with the high GADA group.

The mean of daily alcohol consumption was 13.2 g/day, which is approximately equal to one standard drink. Wine was the most commonly used alcoholic beverage, constituting ∼50% of total alcohol intake (beer, 28% and liquor, 22%).

### Alcohol consumption and type 2 diabetes

Average daily alcohol consumption was inversely associated with the risk of type 2 diabetes (Table 2). Every 5-g of increment in alcohol intake was associated with 5% risk reduction (OR 0.95, 95% CI 0.92–0.99), and the lowest risk was observed in individuals drinking 5–15 g/day (OR 0.56, 95% CI 0.41–0.77). Stratifying the results by sex indicated similar results. The lowest risk was found in individuals who drank alcohol 3–6 times/week (OR 0.49, 95% CI 0.31–0.79), and the risk reduction was confined to consumption of wine (OR/5-g per day, 0.94; 95% CI 0.90–0.99).

### Alcohol consumption and LADA

Overall, alcohol consumption was associated with a reduced risk of LADA; a 6% reduced risk was observed for a 5-g increment in alcohol intake (OR 0.94, 95% CI 0.89–0.99) and a 46% reduced risk was observed in those who drank ≥25 g/day (OR 0.54, 95% CI 0.31–0.94) (Table 3). A tendency of risk reduction was observed in both men (OR 0.95, 95% CI 0.90–1.01) and women (OR 0.90, 95% CI, 0.78–1.05), and the results persisted after additional adjustment for consumption of soft drinks and coffee (OR/5 g per day, 0.95; 95% CI 0.90–1.01); however, the CIs were wide. Stratifying LADA cases by median GADA levels indicated that alcohol was only associated with LADA with low GADA levels (Table 3); a 15% (OR 0.85, 95% CI 0.76–0.94) reduced risk was observed for every 5-g increment in daily alcohol intake and a 77% (OR 0.23, 95% CI 0.10–0.70) risk reduction for the highest consumption group (≥25 g/day). By contrast, there was no association between alcohol intake and LADA with high GADA levels, e.g. OR was estimated at 1.00, 95% CI 0.94–1.06/5-g alcohol per day. The risk reduction in LADA with low GADA levels appeared to be predominantly related to wine (OR 0.84/5 g wine per day, 95% CI 0.71–0.99) rather than beer or liquor consumption. Frequent consumers (≥3 times/week) had a 65% reduced risk (OR 0.35, 95% CI 0.13–0.89), irrespective of the amount consumed.

The results are consistent with the findings of the HUNT prospective study, demonstrating that a reduced risk of alcohol is limited to LADA patients with low GADA levels, HR 0.54, 95% CI 0.29–0.99, vs HR for LADA with high GADA levels, 0.93, 95% CI 0.48–1.80, in those reporting alcohol consumption 1–4 vs <1 time during a 2-week period (Supplementary Table 1, see section on supplementary data given at the end of this article).

### Dose–response analysis

**Figure 1:**
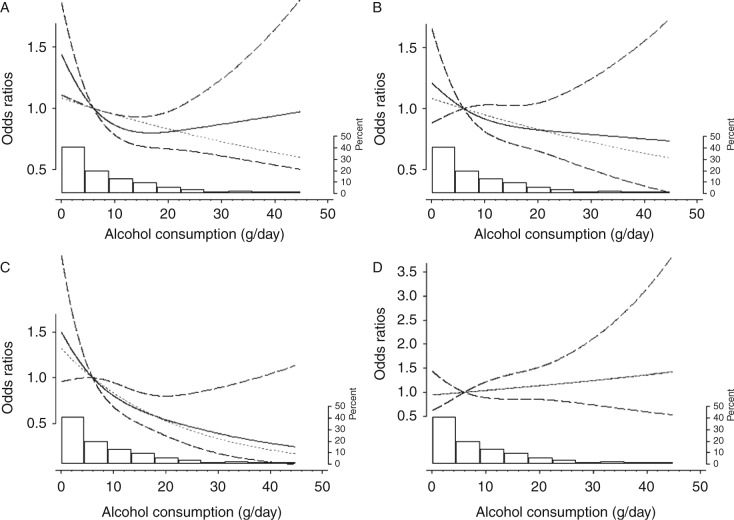
Dose–response odds ratios of type 2 diabetes (A), LADA (B), LADA with low GADA levels (C), and LADA with high GADA levels (D) by alcohol consumption (grams of alcohol per day). Models were adjusted for age, sex, BMI, family history of diabetes, smoking, and education. Results were obtained from ESTRID, 2010–2013. Solid lines represent the spline line; long dashed lines represent the CI of the spline line; and the gray dotted lines represent linear line. The right axis shows the percentage of population distribution across grams of alcohol consumption that corresponds to the histogram.

Restricted cubic spline models were used to explore the potential dose–response relationship between alcohol and diabetes (Fig. 1). For type 2 diabetes, a spline curve fitted better than a linear straight line; a reduced risk was observed for levels of 0.6–23 g of alcohol/day, but there was no further risk reduction above 23 g/day. By contrast, alcohol consumption was dose dependently and linearly associated with the reduced risk of LADA with low GADA levels. For LADA with high GADA levels, if anything, there was a tendency for a positive association.

### Alcohol consumption and levels of GADA, HOMA-IR, and HOMA-β

There was a 10% reduction in HOMA-IR for every 5-g increment in alcohol intake in LADA patients (exp *β*=0.8973, *P*=0.0418) and an 8% reduction in patients with type 2 diabetes (exp *β*=0.9238, *P*=0.0345). With regard to HOMA-β, it appeared to be unrelated to alcohol intake in both LADA (exp *β*=0.8868, *P*=0.1702) and type 2 diabetes (exp *β*=1.0427, *P*=0.2510). Daily alcohol intake was positively associated with GADAs in LADA patients; for every 5-g increment in alcohol intake, a 10% increase in GADA levels was observed (exp *β*=1.1030, *P*=0.0312). Median levels of GADA across categories of non-drinkers, and consumers of 0.01–4.9, 5–14.9, 15–24.9, and ≥25 g alcohol/day were 51, 113, 174, 250, and 250 IU/ml respectively (*P*=0.0267).

### Sensitivity analysis

In an attempt to evaluate the potential influence of recall bias, we performed analysis restricted to patients diagnosed within 3 months before inclusion into the study. The analysis, although based on small numbers, yielded similar results; for LADA with low GADA levels, OR for every 5-g increment in daily alcohol intake was estimated to be 0.62, 95% CI 0.23–1.67.

## Discussion

### Main findings

We could confirm a reduced risk of type 2 diabetes in moderate consumers of alcohol (5, 6, 26). With regard to LADA, alcohol intake was only associated with a reduced risk of LADA with low GADA levels, whereas no association was observed for LADA with high levels of GADA.

Our findings confirm that the LADA concept is heterogeneous; LADA patients with low GADA levels tended to be more type 2-like phenotypically than those with high GADA levels, and the risk factors may also differ (4, 27). It is reported that moderate alcohol consumption improves insulin sensitivity, an effect that may be mediated through inhibiting gluconeogenesis (28), increasing adiponectin plasma levels (29), promoting insulin production by the pancreas (30), and decreasing markers of inflammation (8, 31). In line with this, we observed an inverse association between alcohol and HOMA-IR. The lack of association between alcohol intake and more autoimmune type 1-like LADA indicates that alcohol does not have beneficial effects on the development of autoimmunity. In fact, alcohol consumption was associated with higher levels of GADA. Based on our findings, we hypothesize that alcohol intake through beneficial effects on insulin sensitivity may prevent or delay onset of LADA in the presence of mild autoimmunity, but that an insulin-sensitizing effect may not counter the impact of a more pronounced autoimmune process.

Our findings are in contrast with previous reports of a reduced risk of autoimmune disorders such as rheumatoid arthritis, Graves' hyperthyroidism, and lupus erythematous, related to alcohol intake (9, 10, 11, 32). Still, the link between alcohol and immune response is controversial (33), and in several studies, no association between alcohol intake and these autoimmune diseases was found (34, 35, 36).

Consistent with previous observations (26), we found a U-shaped association between alcohol consumption and type 2 diabetes, with the lowest risk in moderate alcohol consumers (30–40%) (5, 6). In line with most previous studies (1, 26, 37), our findings suggest that the protective effect may primarily be related to consumption of wine. The beneficial effect of wine may be due to the presence of other compounds rather than ethanol in wine, such as polyphenols and hydroxystilbenens, which have anti-oxidative or anti-inflammatory properties (38, 39). It has also been suggested that wine drinking is related to healthier lifestyle behaviors in general, which may reduce the risk of disease (37). Still, our results persisted after adjustment for a number of factors, including BMI, smoking, and education.

### Methodological considerations

These results are based on the largest population-based study of LADA to date. One concern is potential recall bias as information on alcohol was based on retrospective self-reporting. This implies that bias could be introduced if people with diabetes changed their consumption after diagnosis and reported accordingly. To minimize this potential bias, cases received the questionnaire in close proximity to diagnosis with instructions to report their habits before diagnosis. In an attempt to elucidate potential recall bias, we made sensitivity analysis where we restricted the analyses to cases responding to the questionnaire within 3 months of diagnosis. This did not change the results; however, recall bias cannot be ruled out. Importantly, in current dietary guidelines in Sweden, there is no recommendation about changing alcohol consumption for patients with diabetes (40). It should also be noted that our results are in agreement with the findings from prospective studies, with alcohol reported several years before diagnosis, for both type 2 diabetes (1, 5) and LADA (1). However, the number of cases in stratified analyses based on HUNT data is very small and the results are uncertain, especially with regard to abstainers (21). With regard to assessment of IR by HOMA, it should be mentioned that this may not be a valid measure in insulin-treated patients (41). Restricting the analysis to patients without insulin treatment did not change the association between alcohol intake and HOMA-IR (results not shown). Still, HOMA-IR should be regarded as a crude indicator of insulin sensitivity (42). The specificity of GADA assay in this study was 98% (15). False-positive LADA cases could account for some of the similarities between LADA with low GADA levels and type 2 diabetes results, but it is unlikely to explain the lack of association observed between alcohol intake and LADA with high GADA levels.

In conclusion, our data suggest that alcohol consumption may improve insulin sensitivity and reduce the risk of type 2 diabetes and type 2-like LADA alike but has no beneficial effect on diabetes-related autoimmunity.

## Supplementary data

This is linked to the online version of the paper at http://dx.doi.org/10.1530/EJE-14-0403.

## Author contribution statement

All authors contributed to the writing of the manuscript and interpretation of the data, critically reviewed the paper, and read and approved the final manuscript. B Rasouli was responsible for analyzing the data and writing the paper. B Rasouli had access to all data in this study and takes responsibility for the integrity of the data and accuracy of data analysis.

## Supplementary Material

Supplementary Table

## Figures and Tables

**Table 1:** Characteristics of LADA and type 2 diabetes cases and controls in ESTRID, 2010–2013.

**Characteristics**	**Controls**	**Type 2 diabetes**	**LADA**				
All	*P* ^a^	Low GADA levels^b^ (≤ median; 152 IU/ml)	High GADA levels^b^ (> median; 152 IU/ml)	*P* ^c^
No. of individuals	1012	764	250		123	123	
Age, years, mean (s.d.)	59 (13)	63 (10)	60 (12)	<0.0001	62 (12)	57 (12)	0.0015
Men, *n* (%)	468 (46)	462 (60)	138 (55)	<0.0001	72 (58)	64 (52)	0.3694
Low education level, *n* (%)	269 (27)	299 (39)	75 (30)	0.0003	50 (41)	21 (17)	0.0002
BMI, kg/m^2^, mean (s.d.)	26 (4)	31.1 (5)	27.7 (5)	<0.0001	28.5 (5)	26.7 (5)	0.0056
Overweight (25≤BMI), *n* (%)	549 (54)	722 (94)	174 (70)	<0.0001	96 (78)	74 (60)	0.0036
Physically inactive, *n* (%)	608 (61)	568 (75)	162 (66)	0.0104	85 (70)	76 (63)	0.2758
Current smokers, *n* (%)	175 (17)	110 (34)	35 (14)	0.3575	15 (12)	19 (15)	0.7557
With family history of diabetes, *n* (%)	246 (24)	372 (49)	108 (43)	0.1445	52 (42)	52 (42)	1.000
Non-drinkers, *n* (%)^d^	94 (9)	106 (14)	26 (10)	0.1934	16 (13)	9 (7)	0.2048
Insulin treatment, *n* (%)^e^	–	28 (5)	83 (39)	<0.0001	28 (28)	55 (50)	0.0012
Metformin/and or sulfonyl urea treatment, *n* (%)^e^	–	345 (35)	83 (8)	0.0035	35 (14)	48 (20)	0.2430
C-peptide, mean (s.d.), nmol/l^e^	–	1.30 (0.58)	0.76 (0.53)	<0.0001	0.96 (0.56)	0.57 (0.41)	<0.0001
HOMA-IR, mean (s.d.)^e^	–	5.50 (12.77)	5.21 (14.45)	0.7958	6.30 (18.37)	4.09 (8.72)	0.2866
HOMA-β, mean (s.d.)^e^	–	69.53 (35.18)	43.23 (34.27)	<0.0001	53.74 (37.67)	32.39 (26.46)	<0.0001
GADA, median (interquartile range), IU/ml^e^	–	–	152 (12–250)	–	24 (11–120)	250 (203–250)	<0.0001

a *P* for difference between LADA and type 2 diabetes.

b Four LADA cases did not give consent to the record linkage necessary to obtain GADA measurements.

c *P* for difference between LADA with high GADA levels and that with low GADA levels.

d Non-drinkers including abstainers and former drinkers.

e The information is only available for patients with LADA and type 2 diabetes.

**Table 2:** OR of type 2 diabetes in relation to alcohol intake, results from ESTRID, 2010–2013.

	**Cases/controls**	**All**		**Cases/controls**	**Men**	**Cases/controls**	**Women**
OR^a^ (95% CI)	OR^b^ (95% CI)	OR^b^ (95% CI)	OR^b^ (95% CI)
Alcohol intake (g/day)							
Non-drinkers^c^	106/94	1.22 (0.88–1.70)	1.34 (0.89–2.00)	48/34	1.18 (0.65–2.15)	58/60	1.46 (0.84–2.53)
0.01–4.9	305/343	Reference	Reference	138/107	Reference	167/236	Reference
5–14.9	177/340	0.53 (0.42–0.69)	0.56 (0.41–0.77)	127/169	0.58 (0.38–0.88)	50/171	0.49 (0.31–0.79)
15–24.9	83/130	0.62 (0.44–0.86)	0.59 (0.39–0.87)	69/75	0.62 (0.37–1.02)	14/55	0.46 (0.22–0.98)
≥25	93/105	0.76 (0.54–1.06)	0.58 (0.38–0.88)	80/83	0.51 (0.31–0.84)	13/22	1.26 (0.49–3.26)
Alcohol intake (per 5 g/day)		0.98 (0.95–1.01)	0.95 (0.92–0.99)		0.96 (0.92–0.99)		0.94 (0.85–1.04)
Frequency of alcohol intake^d^							
≤1 time/month	147/159	Reference	Reference	56/50	Reference	91/109	Reference
2–3 times/month	186/230	0.78 (0.58–1.07)	0.80 (0.55–1.17)	109/85	0.88 (0.48–1.60)	77/145	0.79 (0.47–1.32)
1–2 times/week	199/323	0.54 (0.39–0.73)	0.56 (0.38–0.82)	145/173	0.61 (0.35–1.06)	54/150	0.47 (0.25–0.86)
3–6 times/week	94/169	0.40 (0.27–0.59)	0.49 (0.31–0.79)	77/101	0.51 (0.27–0.97)	17/68	0.43 (0.17–1.07)
≥1 time/day	32/37	0.46 (0.24–0.88)	0.67 (0.30–1.46)	27/25	0.55 (0.21–1.45)	5/12	1.04 (0.24–4.51)
Alcoholic beverages	658/918			414/434		244/484	
Beer (per 5 g/day)		1.00 (0.96–1.04)	1.01 (0.96–1.06)		1.02 (0.97–1.07)		1.02 (0.71–1.48)
Wine (per 5 g/day)		0.96 (0.92–1.00)	0.94 (0.90–0.99)		0.95 (0.89–1.00)		0.92 (0.83–1.03)
Liquor (per 5 g/day)		1.01 (0.96–1.05)	1.00 (0.94–1.05)		0.99 (0.94–1.05)		1.18 (0.79–1.78)

a Adjusted for age and sex.

b Adjusted for age, sex, BMI, family history of diabetes, smoking, and education.

c Non-drinkers including abstainers and former drinkers.

d Analyses were run only in alcohol drinkers; additional adjustment for total alcohol intake.

**Table 3:** OR of LADA in relation to alcohol intake, results from ESTRID, 2010–2013.

	**All**			**LADA low GADA levels** (≤ median; 152 IU/ml)		**LADA high GADA levels** (> median; 152 IU/ml)	
Cases/controls	OR^a^ (95% CI)	OR^b^ (95% CI)	Cases/controls^c^	OR^b^ (95% CI)	Cases/controls^c^	OR^b^ (95% CI)
Alcohol intake (g/day)							
Non-drinkers^d^	26/94	0.98 (0.59–1.61)	0.95 (0.56–1.57)	16/94	0.97 (0.51–1.84)	9/94	0.92 (0.42–2.01)
0.01–4.9	94/343	Reference	Reference	54/343	Reference	39/343	Reference
5–14.9	74/340	0.73 (0.52–1.03)	0.75 (0.52–1.08)	36/340	0.60 (0.37–0.97)	38/340	0.95 (0.58–1.56)
15–24.9	35/130	0.87 (0.56–1.37)	0.79 (0.50–1.26)	11/130	0.42 (0.21–0.86)	23/130	1.33 (0.74–2.38)
≥25	21/105	0.59 (0.35–1.02)	0.54 (0.31–0.94)	6/105	0.23 (0.10–0.57)	14/105	1.01 (0.50–2.02)
Alcohol intake (per 5 g/day)		0.95 (0.90–1.01)	0.94 (0.89–0.99)		0.85 (0.76–0.94)		1.00 (0.94–1.06)
Frequency of alcohol intake^e^							
≤1 time/month	42/159	Reference	Reference	27/159	Reference	14/159	Reference
2–3 times/month	62/230	0.98 (0.63–1.53)	0.98 (0.62–1.55)	34/230	0.83 (0.46–1.48)	28/230	1.40 (0.70–2.80)
1–2 times/week	85/323	0.89 (0.58–1.37)	0.84 (0.54–1.31)	35/323	0.62 (0.33–1.16)	49/323	1.44 (0.74–2.78)
3–6 times/week	25/169	0.47 (0.27–0.83)	0.48 (0.27–0.87)	23/206^f^	0.35 (0.13–0.89)	11/206^f^	1.06 (0.48–2.35)
≥1 time/day	10/37	0.71 (0.28–1.79)	0.68 (0.26–1.77)	–	–	–	–
Alcoholic beverages	224/918			107/918		114/918	
Beer (per 5 g/day)		1.03 (0.98–1.08)	1.03 (0.99–1.09)		0.83 (0.62–1.10)		1.03 (0.97–1.09)
Wine (per 5 g/day)		0.96 (0.90–1.03)	0.95 (0.89–1.02)		0.84 (0.71–0.99)		0.95 (0.85–1.06)
Liquor (per 5 g/day)		0.99 (0.91–1.07)	0.97 (0.88–1.07)		0.88 (0.72–1.08)		1.02 (0.93–1.12)

a Adjusted for age and sex.

b Adjusted for age, sex, BMI, family history of diabetes, smoking, and education.

c Four LADA cases did not give consent to use their clinical information, including GADA measurements.

d Non-drinkers including abstainers and former drinkers.

e Analyses were run only in alcohol drinkers; additional adjustment for total alcohol intake.

f Two high-frequent alcohol intake categories were combined because of small numbers (daily alcohol drinkers and who drank 3–6 times/week).
