# Supplementary material for: Alcohol and the risk for latent autoimmune diabetes in adults: results based on Swedish ESTRID study
Source: Eur J Endocrinol. 2014 Jul 22;171(5):535–43. doi: 10.1530/EJE-14-0403 (PMC4190680; doi:10.1530/EJE-14-0403)
Supplement: Supplementary Table [file supp_171_5_535__index.html]

Alcohol and the risk for latent autoimmune diabetes in adults: results based on Swedish ESTRID study — Alcohol and LADA — Supplementary Table 

# Alcohol and the risk for latent autoimmune diabetes in adults: results based on Swedish ESTRID study

## Supplementary Table

**Files in this Data Supplement:**

- Supplementary Table 1 - (PDF 92 KB)
