# Supplementary material for: Alcohol and the risk for latent autoimmune diabetes in adults: results based on Swedish ESTRID study
Source: Eur J Endocrinol. 2014 Jul 22;171(5):535–43. doi: 10.1530/EJE-14-0403 (PMC4190680; doi:10.1530/EJE-14-0403)
Supplement: Supplementary Table [file supp_EJE-14-0403_Supplementary_table_1.pdf]

Supplementary Table 1. HR of autoimmune diabetes in adults stratified by median GADA levels, in relation to frequency of alcohol intake, HUNT study, 1984-2008

		Autoimmune diabetes in adults			
			Low GADA (≤median)*		*High GADA (>median*)*
	Person-year	No. cases	HR† (95% CI)	No. cases	HR† (95% CI)
**Frequency of alcohol intake during the last 14 days**					
Abstainers	103,167	5	0.30 (0.10-0.86)	11	1.63 (0.74-3.57)
<1 time	353,919	39	Reference	20	Reference
1-4 times	451,268	19	0.54 (0.29-0.99)	22	0.93 (0.48-1.80)
≥5 times	44,113	2	0.48 (0.11-2.07)	4	1.24 (0.35-4.33)

* Median of GADA=0.16 index value in relation to standard serum, † HR controlled for age and sex, BMI, Smoking, family history of diabetes, education and physical activity
